# Design and Evaluation of a Multicomponent Herbal Nanogel for Mosquito Repellency: An Integrated In Silico Experimental Approach Against *Aedes aegypti* and *Anopheles stephensi*

**DOI:** 10.1155/jotm/6513704

**Published:** 2026-08-03

**Authors:** Mohsen Safaei, Alireza Sanei-Dehkordi, Navid Alinejad, Mahsa Fallahi, Mahmoud Osanloo

**Affiliations:** ^1^ Department of Medical Biotechnology, School of Advanced Technologies in Medicine, Fasa University of Medical Sciences, Fasa, Iran, fums.ac.ir; ^2^ Emerging and Re-Emerging Infectious Diseases Research Center, Hormozgan Health Institute, Hormozgan University of Medical Sciences, Bandar Abbas, Iran, hums.ac.ir; ^3^ Department of Biology and Control of Disease Vectors, Faculty of Health, Hormozgan University of Medical Sciences, Bandar Abbas, Iran, hums.ac.ir; ^4^ Department of Public Health, School of Health, Fasa University of Medical Sciences, Fasa, Iran, fums.ac.ir; ^5^ Department of Medical Nanotechnology, School of Advanced Technologies in Medicine, Fasa University of Medical Sciences, Fasa, Iran, fums.ac.ir

**Keywords:** DEET alternative, herbal nanogel, molecular docking, mosquito repellency

## Abstract

This study describes the development and characterization of a hydroxypropyl methylcellulose (HPMC)–based nanogel. The nanogel coencapsulates thymol, carvacrol, and cinnamaldehyde to enhance mosquito repellency. These phytochemicals were selected for their potential complementary and multimodal neurotoxic and olfactory‐disrupting effects. The nanogel was synthesized by spontaneous emulsification, followed by polymeric gelation. Its physicochemical properties were assessed with dynamic light scattering (DLS), zeta potential, rheological measurements, and stability tests. The nanogel showed an optimal size (177 ± 6 nm; PDI: 0.17 ± 0.02; SPAN: 0.96), robust colloidal stability, and pseudoplastic flow behavior. This is consistent with the Carreau–Yasuda model. Repellency assays showed complete protection for 410 ± 45 min against *Anopheles stephensi* and 210 ± 30 min against *Aedes aegypti.* Specifically, the nanogel was statistically superior to 40% DEET against *Anopheles stephensi*, while providing comparable (noninferior) protection against *Aedes aegypti*. Molecular docking showed strong binding affinities of thymol and carvacrol to mosquito odorant‐binding proteins providing mechanistic support for the potential complementary activity of the multicomponent formulation. The formulation maintained physical and rheological stability for 6 months. There was no phase separation or syneresis. These findings show this nanogel’s promise as an effective natural mosquito repellent.

## 1. Introduction

Mosquito‐borne diseases like malaria, dengue, chikungunya, and Zika remain urgent global health concerns, especially across tropical and subtropical regions [1, 2]. *Aedes aegypti* and *Anopheles stephensi* stand out as pivotal mosquito vectors in the battle to contain these illnesses*. Ae. aegypti* spreads dengue, chikungunya, Zika, and yellow fever in both urban and rural areas [3]. Meanwhile, *An. stephensi*, South Asia’s main malaria vector, has dramatically expanded its reach—fueling resurgences, such as Djibouti’s jump from 2.5 to 97.6 malaria cases per 1000 people between 2013 and 2020 [4, 5].

Effective vector control is essential because vaccines and therapeutics are limited. Current strategies include chemical insecticides, biological control with predators or pathogens, and genetic approaches using sterile or GMO mosquitoes. Topical repellents provide personal protection. However, synthetic repellents like DEET can cause skin irritation and possible neurotoxicity and require frequent reapplication [6, 7].

Consequently, there is an increasing interest in plant‐derived bioactive constituents as safer and more sustainable alternatives. Among these, cinnamaldehyde, thymol, and carvacrol were prioritized in this study due to their high biological activity at relatively low concentrations, favorable dermal safety profiles, and well‐documented efficacy against various mosquito species. Unlike many volatile terpenes that exhibit predominantly single‐target effects, these three compounds were deliberately selected to exploit their distinct yet complementary neuro‐olfactory mechanisms [8, 9].

The rationale for this multicomponent approach lies in the diverse modes of action of the selected phytochemicals. While each compound demonstrates significant individual activity, their mechanistic diversity suggests potential additive or complementary effects, with possible synergistic interactions in the combined formulation [10, 11]. Specifically, thymol primarily interferes with octopaminergic and GABAergic signaling pathways, leading to neural inhibition and paralysis [12, 13]. In contrast, carvacrol—despite its structural similarity to thymol—modulates neuronal ion channels and induces membrane depolarization, resulting in excitatory neurotoxicity [14, 15].

Complementing these neural effects, cinnamaldehyde targets TRPA1 and ionotropic receptors involved in olfactory signaling, thereby disrupting odor detection and functioning as both a repellent and a toxicant [16, 17]. By simultaneously impacting neural transmission (via thymol and carvacrol) and odor perception (via cinnamaldehyde), this multitarget strategy potentially enhances overall efficacy while reducing the likelihood of resistance development. Such a combination of phytochemicals with distinct molecular targets has been shown to yield superior bioactivity compared to single‐compound formulations [17, 18].

Despite their efficacy, these compounds are limited by high volatility and instability. Nanotechnology improves compound stability and allows controlled release. Nanogels, which are hydrogel‐based nanocarriers, extend repellency duration, improve skin adhesion, and reduce how often products must be reapplied [19, 20]. Compared with other systems (nanoemulsions, chitosan particles, solid lipid nanoparticles), nanogels have clear advantages. Their 3D hydrophilic polymer network gives better encapsulation stability, prolongs release, and improves skin adhesion. These traits are essential for topical repellents. Also, nanogels are nongreasy and biocompatible, and they retain volatile essential oils better than nanoemulsions or lipid systems. This improves efficacy and user compliance [21, 22].

In addition to experimental evaluation, molecular docking provides valuable mechanistic insight into repellent activity. Odorant‐binding proteins (OBPs) are crucial in mosquito olfaction, mediating host‐seeking and odor perception [23, 24]. Plant‐derived compounds that strongly bind OBPs can block olfactory signaling and serve as eco‐friendly alternatives to synthetic repellents [25, 26].

In our previous study, we prepared and tested hydroxypropyl methylcellulose (HPMC)‐based nanogels containing thymol, carvacrol, and cinnamaldehyde (each at 3% w/v) against *Ae. aegypti* [27]. Each nanogel was protected longer (250, 240, and 200 min) than commercial DEET (190 min). This highlights the HPMC‐nanogel’s potential for use as a botanical repellent. We hypothesized that a multicomponent nanogel containing all three phytochemicals would enhance efficacy through their combined complementary effects. Here, we (i) make a single nanogel coencapsulating all three actives, (ii) test efficacy against *Ae. aegypti* and *An. stephensi* for broad‐spectrum action, and (iii) add molecular docking studies to reveal how these compounds bind to mosquito OBPs. This supports a mechanistic explanation of repellency.

## 2. Materials and Methods

### 2.1. Materials

Thymol, cinnamaldehyde, carvacrol, and Tween 20 were purchased from Merck Chemical Company (Germany). HPMC was purchased from Sigma‐Aldrich Company (USA). Additionally, a commercial 40% DEET formulation, a commonly used repellent in Iran, was supplied by Reyhan Naghsh Jahan Pharmaceutical Company (Iran).

### 2.2. Preparation and Characterization of the Nanogel

The nanogel was developed through a two‐step process involving initial nanoemulsion formation followed by polymeric gelation.

**Step 1.**

Optimization and Preparation of the Nanoemulsion

An oil‐in‐water nanoemulsion was prepared by spontaneous emulsification [2]. The active component phase consisted of thymol, carvacrol, and cinnamaldehyde, each at 1% w/v, for a total active content of 3% w/v. This total concentration was selected to ensure optimal repellent efficacy while maintaining sensory acceptability and controlling the final product’s odor intensity.

The surfactant concentration was systematically optimized. Formulations containing Tween 20 at 4%, 5%, 6%, and 7% w/v were prepared and evaluated. The active compounds and surfactant mixture were stirred at 2000 rpm for 10 min at room temperature. Distilled water was added dropwise to the oil–surfactant mixture under continuous stirring (2000 rpm for 40 min). The formulation containing 6% Tween 20 was selected as the optimal composition, as it produced a nanoemulsion with the smallest particle size, lowest polydispersity index (PDI), and narrowest particle size distribution (SPAN).

**Step 2.**

Conversion to the Nanogel

The optimized nanoemulsion was converted into a nanogel using HPMC as the gelling agent. HPMC was evaluated at three concentrations (1.0%, 1.5%, and 2.0% w/v) to determine the optimal gelling capacity. The polymer was gradually incorporated into the nanoemulsion under constant stirring (2000 rpm) at room temperature to prevent aggregation. The mixture was subsequently stirred overnight at ambient temperature to ensure complete polymer hydration and formation of a homogeneous nanogel.

The 1.5% HPMC concentration was identified as optimal, providing desirable viscosity. A blank nanogel (without active compounds) was prepared following the identical protocol for use as a control in subsequent analyses.

Since the 1.0% HPMC formulation was too fluid and the 2.0% formulation formed a semisolid gel, only the 1.5% HPMC nanogel was selected for subsequent rheological characterization. The flow behavior of the nanogel and the blank gel (1.5% HPMC) was assessed using a rotational rheometer (MCR‐302, Anton Paar, Austria), with viscosity measured over shear rates ranging from 0.1 to 100 s^−1^. Moreover, their stability was evaluated for 6 months under two storage conditions: refrigerated (4°C) and ambient temperature (25 ± 2°C). Samples were periodically examined (initially and after 1, 3, and 6 months) for visual appearance, color change, phase separation, and sedimentation.

### 2.3. Mosquito‐Repellent Bioassays

The mosquito‐repellent efficacy of the nanogels was evaluated using the standard World Health Organization (WHO) arm‐in‐cage protocol [28]. All methods were performed in accordance with the relevant WHO and internal guidelines and regulations. In compliance with Iranian national regulations, the study was exempt from clinical trial classification. Ethical approval was obtained from the Ethics Committee of Fasa University of Medical Sciences (IR.FUMS.REC.1404.020). A 22‐year‐old female volunteer provided written informed consent after receiving a full explanation of the experimental procedures. Test mosquitoes consisted of 5–7‐day‐old, non‐blood‐fed, nulliparous female *Ae. aegypti* (Bandar Abbas strain) and *An. stephensi* (Bandar Abbas strain) sourced from the Culicidae Insectary of the School of Public Health, Hormozgan University of Medical Sciences, Iran. These mosquitoes were reared under controlled conditions (27 ± 2°C, 70 ± 5% relative humidity, and a 12‐h light/dark cycle) in 40 × 40 × 40‐cm cages.

Before testing, adult female *Ae. aegypti* or *An. stephensi* mosquitoes were deprived of a 10% sucrose solution for 14 h. The volunteer’s forearm was disinfected with 70% ethanol, and a defined rectangular area (8 × 12.5 cm, 100 cm^2^) on the inner surface was uniformly treated with 1 g of the test samples (nanogel, blank gel, and DEET 40%). This corresponded to an application rate of 10 mg of formulation per cm^2^; for the active compound‐loaded nanogel, this equated to 0.3 mg of the active compounds per cm^2^ (i.e., 30 mg of thymol, carvacrol, and cinnamaldehyde in total), given its 3% (w/w) loading. The same amount (1 g) of the blank nanogel (without active compounds) and a 40% DEET solution (as a positive control) were applied separately in the same manner. After a 5‐min drying period, the treated forearm was exposed to a cage containing 250 adult female mosquitoes for 3 min. The exposure was repeated at 30 min intervals until the first confirmed mosquito landing occurred. The elapsed time from application to the initial landing was recorded as the complete protection time.

The biological evaluation was designed as an exploratory pilot study involving a single healthy volunteer. Due to the nature of the assay and the use of a single volunteer, the tests were not blinded. Each treatment was evaluated in three independent technical replicates using the single volunteer. For each replicate, a fresh batch of 250 unfed female mosquitoes was used to eliminate potential behavioral conditioning or prior exposure effects. Results were expressed as mean ± standard deviation (SD). To avoid potential carryover effects, each treatment was evaluated on a separate experimental day, allowing sufficient time for the treated skin to fully dry and for any residual odor from previous applications to dissipate. In practice, experiments were conducted at approximately 1‐week intervals under comparable environmental conditions. Data normality was assessed using the Shapiro–Wilk test. The *An. stephensi* data were normally distributed, whereas the *Ae. aegypti* data did not follow a normal distribution. Consequently, one‐way ANOVA followed by Tukey’s HSD multiple comparisons test was used for *An. stephensi*. For *Ae. aegypti*, the Kruskal–Wallis test was performed, followed by Dunn’s multiple comparisons test with multiplicity‐adjusted *p* values to account for multiple pairwise comparisons. Results are presented as mean ± SD, and statistical significance was defined as *p* < 0.05.

### 2.4. In Silico Study

In this study, four ligands were selected for molecular docking analysis, including three plant‐derived compounds, carvacrol, thymol, and cinnamaldehyde, and one synthetic repellent, N,N‐diethyl‐meta‐toluamide (DEET), used as a reference. The 3D structures of the ligands were retrieved from the PubChem compound database (https://pubchem.ncbi.nlm.nih.gov/) and structurally optimized using Avogadro software (Version 1.2, https://avogadro.cc/) via energy minimization.

Five OBPs or odorant receptors (ORs) from *Ae. aegypti*, *An*. *gambiae*, and *An. stephensi* were selected as molecular targets. Due to the high similarity of OBPs across species, these targets were considered for study. Three of these protein structures were obtained from the Protein Data Bank (PDB, https://www.rcsb.org/), including AaegOBP1 (PDB ID: 3K1E), AgamOBP1 (PDB ID: 3N7H), and AgamOBP5 (PDB ID: 8BXW). The remaining two proteins—AgamOR1 and AsteOBP1—were modeled using the SWISS‐MODEL homology modeling server (https://swissmodel.expasy.org/), based on amino acid sequences retrieved from the NCBI Protein Database (https://www.ncbi.nlm.nih.gov/protein/).

Binding site prediction for the modeled structures was performed using PrankWeb: Ligand Binding Site Prediction Server (https://prankweb.cz/), which applies machine learning algorithms to predict ligand‐accessible pockets. For crystal structures in the PDB, ligand‐binding sites were also defined based on natural ligands and cavity detection using Molegro Virtual Docker (MVD) software.

Molecular docking simulations were conducted using Molegro Virtual Docker (MVD). The docking search space was defined around the predicted active site, and default docking parameters were used. Ligand‐receptor binding affinities were evaluated using MolDock Score and Re‐rank Score. Additionally, hydrogen bond energies were computed to assess interaction types. Protein–ligand interactions were visualized and analyzed using BIOVIA Discovery Studio Visualizer, allowing for identification of key residues involved in hydrogen bonding, hydrophobic contacts, π–π stacking, and van der Waals interactions.

### 2.5. Sequential Docking Modeling of Selected Compounds With OBP1

To investigate the simultaneous binding pattern and evaluate the structural basis for the combined effects, two OBP1 proteins were selected as representative targets for the study: OBP1 from *Ae*. *aegypti* and a modeled OBP1 from *An*. *stephensi*. These two targets were considered representative models for investigating ligand binding behavior due to their functional similarity and biological importance. Docking of the compounds was performed in a sequential manner: each ligand was initially docked separately to the target protein, and the most stable complex was selected; then, the second ligand was docked onto the complex formed by the first ligand and protein. This approach enabled investigation of simultaneous binding modes, the degree of spatial overlap, and the potential for complementary occupancy of distinct regions of the binding cavity.

## 3. Results

The surfactant concentration was optimized to obtain a stable, uniform nanoemulsion carrier. As shown in Table 1, the formulation containing 6% Tween 20 resulted in a monodisperse nanoemulsion with favorable characteristics (particle size: 177 ± 6 nm; PDI: 0.17 ± 0.02; SPAN: 0.96). Lower surfactant concentrations led to biphasic systems, while 7% Tween 20 produced larger droplets (250 ± 8 nm) with increased polydispersity.

**TABLE 1:** Effect of Tween 20 concentration on the size characteristics of oil‐in‐water nanoemulsions containing a 3% (w/v) active oil phase (1:1:1 ratio of thymol, carvacrol, and cinnamaldehyde).

Tween 20 (% w/v)	Particle size (nm)	SPAN	PDI
4%	Biphasic		
5%	Biphasic		
6%	177	0.96	0.17
7%	250	0.96	0.45

**FIGURE 1:**
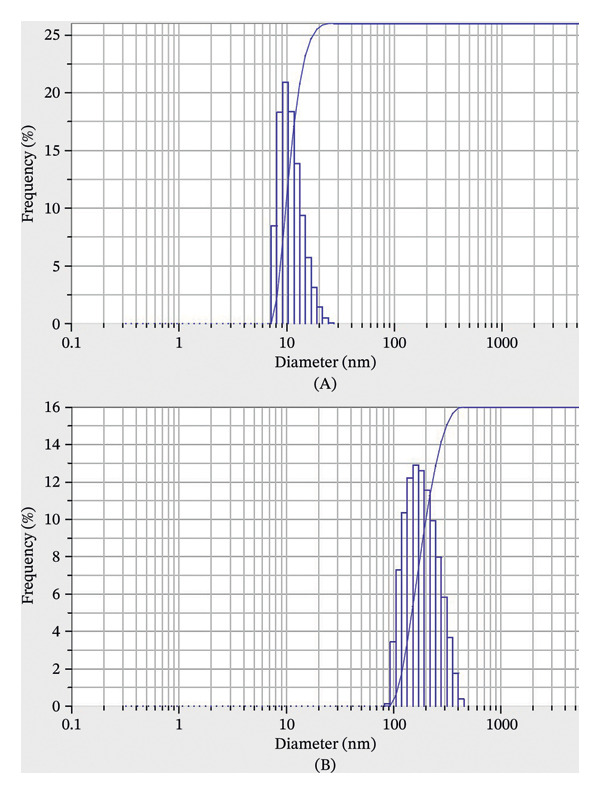
Dynamic light scattering (DLS) size distribution profiles of (A) blank nanoemulsion and (B) thymol–carvacrol–cinnamaldehyde‐loaded nanoemulsion.

DLS analysis (Figure 1) confirmed that both the blank nanoemulsion (10 ± 2 nm; PDI: 0.14; SPAN: 0.66) and the active‐loaded nanoemulsion maintained stable colloidal dispersions, with particle sizes below 200 nm, PDI < 0.7, and SPAN < 1. These results demonstrate that the optimized formulation meets the physicochemical criteria for effective topical application [29].

**FIGURE 2:**
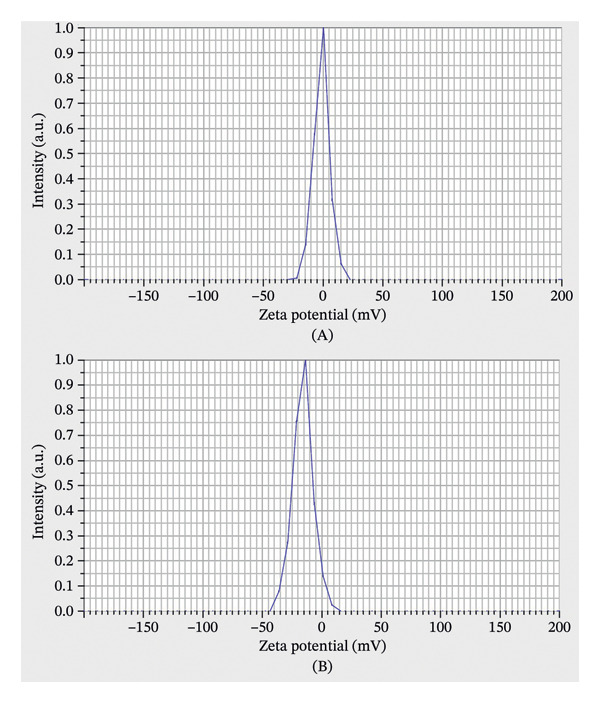
Zeta potential distributions of (A) blank nanoemulsion and (B) thymol–carvacrol–cinnamaldehyde‐loaded nanoemulsion.

Zeta potential measurements (Figure 2) showed a near‐neutral charge for the blank gel (−1.5 ± 0.5 mV), while the nanogel exhibited a stronger negative charge (−16 ± 3 mV), confirming enhanced colloidal stability.

**FIGURE 3:**
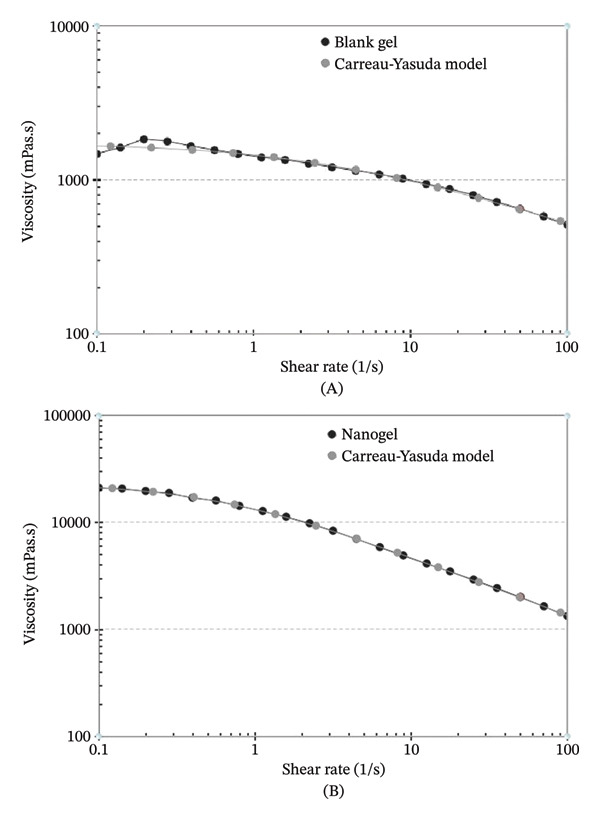
Rheological profiles showing the viscosity of the (A) blank gel and (B) nanogel as a function of shear rate.

Both samples exhibited pronounced shear‐thinning behavior, with viscosity decreasing as the shear rate increased (Figure 3). The experimental data fitted well to the Carreau–Yasuda model, confirming pseudo‐plastic fluid characteristics typical of non‐Newtonian gels. This flow behavior facilitates easy application under shear stress (e.g., during skin spreading) while maintaining structural integrity at rest.

Stability evaluation over 6 months (at 1, 3, and 6 months) showed no visible signs of phase separation, precipitation, or syneresis in either formulation at 4°C or at ambient temperature. The nanogel maintained a consistent appearance and homogeneity throughout the storage period. These findings confirm that the HPMC‐based nanogel exhibits sustained physical and rheological stability, making it suitable for topical repellent formulations.

The ATR‐FTIR spectra of thymol, carvacrol, cinnamaldehyde, blank nanogel, and nanogel (thymol–carvacrol–cinnamaldehyde‐loaded nanogel) were analyzed to verify the presence of characteristic functional groups and to assess molecular interactions within the nanogel matrix.

**FIGURE 4:**
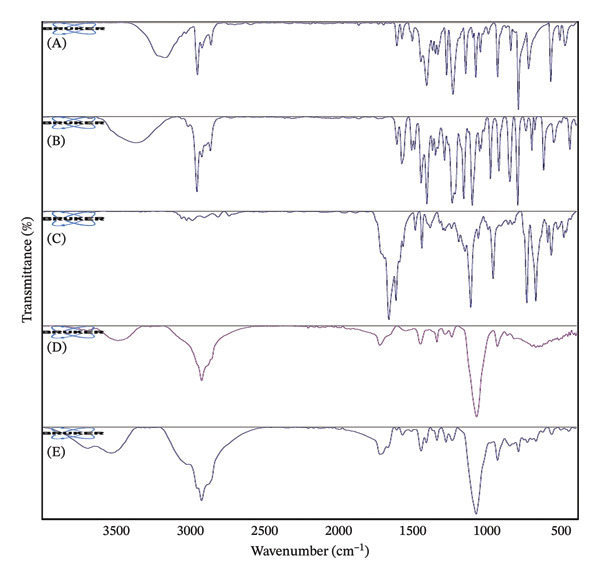
Attenuated total reflection–Fourier transform infrared (ATR‐FTIR) spectra of (A) thymol, (B) carvacrol, (C) cinnamaldehyde, (D) blank gel, and (E) nanogel (thymol–carvacrol–cinnamaldehyde‐loaded).

Figure 4A: Thymol exhibited a broad O–H stretching band at 3178 cm^−1^ and aromatic C=C stretching between 1584 and 1619 cm^−1^. Peaks at 1378 and 1359 cm^−1^ correspond to isopropyl methyl group stretching, while signals at 1004–1057 cm^−1^ and a prominent band at 803 cm^−1^ indicate para‐substitution and ring vibrations, respectively. Additional peaks at 3035, 2957, 2926, and 2867 cm^−1^ were ascribed to =C–H and aliphatic C–H stretching modes.

Figure 4B: Carvacrol displayed O–H stretching at 3373 cm^−1^ and aromatic C=C stretching around 1588–1620 cm^−1^. The bands at 2869 and 2926 cm^−1^ were assigned to symmetric and asymmetric –CH_2_– stretching, with a notable out‐of‐plane aromatic C–H bending observed at 811 cm^−1^. Other peaks at 1249, 1173, 1115, 993, and 864 cm^−1^ confirmed the presence of substituted phenolic structures.

Figure 4C: Cinnamaldehyde showed a strong C=O stretching band at 1671 cm^−1^ and conjugated C=C stretching at 1623 cm^−1^, with additional aromatic C=C signals between 1449 and 1575 cm^−1^. Aromatic and aliphatic C–H stretching appeared at 3060, 3027, and 2988 cm^−1^, while the band at 1160 cm^−1^ was attributed to aromatic C–H bending.

Figure 4D: The blank nanogel spectrum revealed a broad O–H band at 3483 cm^−1^ (Tween 20 and water hydrogen bonding), a C–H stretching peak at 2924 cm^−1^, and a strong C=O band at 1733 cm^−1^ (Tween 20 ester groups). The band at 1085 cm^−1^ corresponded to C–O stretching, with minor shifts indicating interactions between Tween 80 and HPMC.

Figure 4E: The nanogel spectrum presented a broad absorption range from 3529 to 3682 cm^−1^, confirming extensive hydrogen bonding among the components. The C–H stretching peak at 2924 cm^−1^, a carbonyl band at 1732 cm^−1^, and a C–O stretching signal at 1088 cm^−1^ confirmed the successful incorporation of thymol, carvacrol, and cinnamaldehyde into the nanogel. Major peaks from each constituent affirmed their compatibility and stable integration into the nanogel matrix.

**FIGURE 5:**
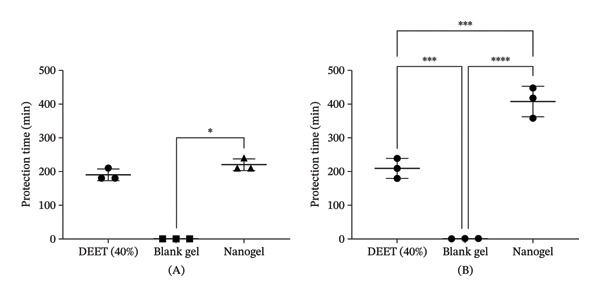
Protection times of the developed nanogel, DEET (40%), and blank gel against (A) *Ae. aegypti* and (B) *An. stephensi*. Data are presented as mean ± SD with individual observations (*n* = 3 independent technical replicates from a single volunteer). Statistical analysis was performed using the Kruskal–Wallis test followed by Dunn’s multiple comparisons test with multiplicity‐adjusted *p* values for *Ae. aegypti*, and one‐way ANOVA followed by Tukey’s HSD multiple comparisons test for *An. stephensi*. Statistical significance is indicated as follows: ^∗^ *p* < 0.05, ^∗∗∗^ *p* < 0.001, and ^∗∗∗∗^ *p* < 0.0001.

As illustrated in Figure 5, the statistical analysis of protection time yielded different results for the two mosquito species, depending on their data distributions. Given the exploratory pilot design involving a single volunteer, inferential statistical analyses were performed to facilitate within‐study comparisons; therefore, the statistical findings should be interpreted with appropriate caution. For *An. stephensi*, one‐way ANOVA revealed significant differences in protection time among the treatment groups (*p* < 0.0001). The mean protection times were 210 ± 30 min for DEET (40%), 410 ± 45 min for the nanogel, and 0.7 ± 0.6 min for the blank gel. Post hoc Tukey’s HSD multiple comparisons test demonstrated that all pairwise comparisons were statistically significant, including DEET (40%) versus blank gel (adjusted *p* = 0.0005), DEET (40%) versus nanogel (adjusted *p* = 0.0006), and blank gel versus nanogel (adjusted *p* < 0.0001).

Conversely, for *Ae. aegypti*, the nonparametric Kruskal–Wallis test revealed a significant overall difference among the three treatment groups (exact *p* = 0.0107). The mean protection times were 190 ± 17 min for DEET (40%), 220 ± 17 min for the nanogel, and 0.7 ± 0.6 min for the blank gel. Pairwise comparisons using Dunn’s multiple comparisons test with multiplicity‐adjusted *p* values showed that the nanogel was associated with significantly longer protection than the blank gel (adjusted *p* = 0.028). However, no significant differences were observed between DEET (40%) and the blank gel (adjusted *p* = 0.379) or between DEET (40%) and the nanogel (adjusted *p* = 0.853).

### 3.1. In Silico Results

**FIGURE 6:**
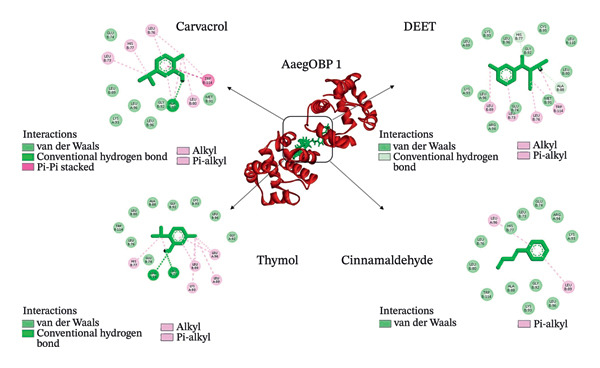
Two‐dimensional and three‐dimensional representations of the docking poses of DEET, carvacrol, thymol, and cinnamaldehyde within the binding site of *Ae. aegypti* odorant‐binding protein 1 (AaegOBP1).

**FIGURE 7:**
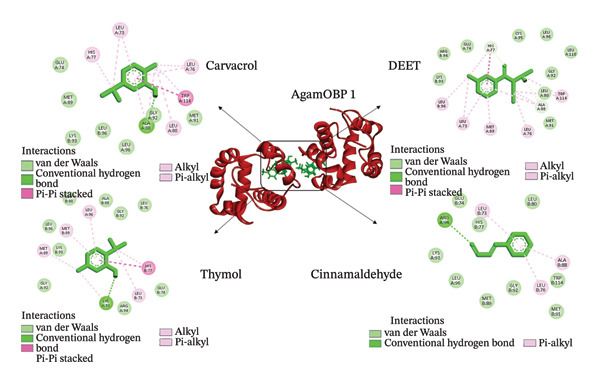
Two‐dimensional and three‐dimensional representations of the docking poses of DEET, carvacrol, thymol, and cinnamaldehyde within the binding site of *Anopheles gambiae* odorant‐binding protein 1 (AgamOBP1).

**FIGURE 8:**
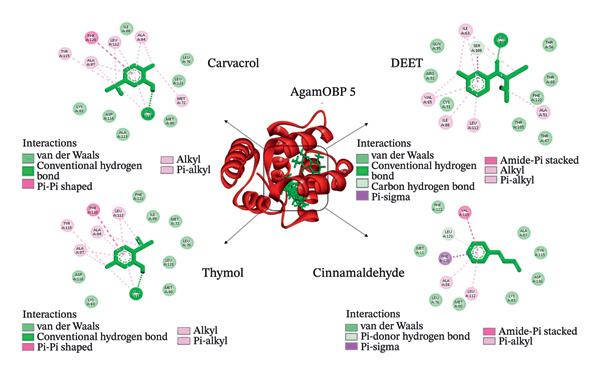
Two‐dimensional and three‐dimensional representations of the docking poses of DEET, carvacrol, thymol, and cinnamaldehyde within the binding site of *Anopheles gambiae* odorant‐binding protein 5 (AgamOBP5).

**FIGURE 9:**
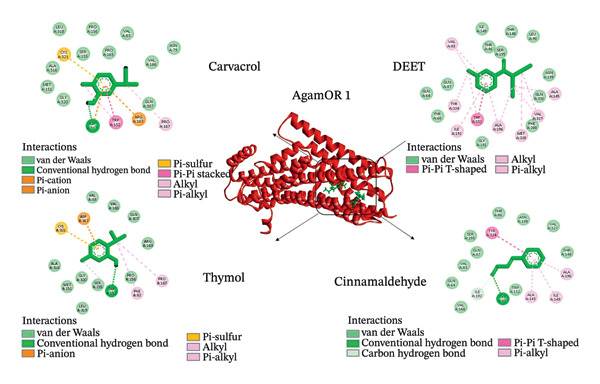
Two‐dimensional and three‐dimensional representations of the docking poses of DEET, carvacrol, thymol, and cinnamaldehyde within the binding site of the modeled *Anopheles gambiae* odorant receptor 1 (AgamOR1).

**FIGURE 10:**
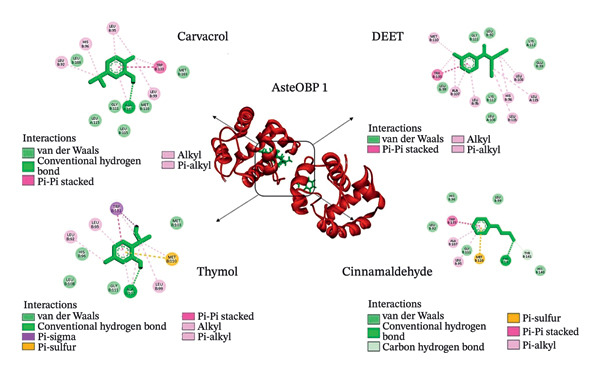
Two‐dimensional and three‐dimensional representations of the docking poses of DEET, carvacrol, thymol, and cinnamaldehyde within the binding site of the modeled *An*. *stephensi* odorant‐binding protein 1 (AsteOBP1).

Table 2 shows that the docking scores suggest that the chemical compound DEET has lower (more negative) binding energies with most receptors, especially AgamOBP1 (−84.7 kcal/mol) and AaegOBP1 (−85.8 kcal/mol). However, the two plant‐based chemicals, carvacrol and thymol, also have good binding affinities to the receptors, with only minor differences from DEET in some cases. For example, the MolDock score for thymol with AaegOBP1 is −78.7 kcal/mol, whereas that for carvacrol is −78.0 kcal/mol. Investigating chemical interactions in images shows that amino acid residues such as LEU 73, HIS 77, and TRP 114 are often implicated in the binding of both plant compounds and DEET to most receptors. The AaegOBP1 and AgamOBP1 receptors have a lot of these residues. Also, residues such as Leu, Ala, Met, Tyr, Phe, and Asp are often found in the binding pockets of both modeled receptors, AgamOR1 and AsteOBP1 (Figures 6–10). This suggests that there are conserved areas in the olfactory proteins of mosquitoes. This demonstrates the potential utility of creating drugs or repellents that simultaneously act on multiple receptors. Furthermore, unlike DEET, which primarily interacts through van der Waals forces and π–alkyl interactions, plant compounds are more involved in hydrogen bonding. For example, carvacrol and thymol formed hydrogen bonds with AaegOBP1 and AaegOBP1 with energies of about −4.5 and −2.9 kcal/mol, respectively, higher than the other ligands.

**TABLE 2:** Molecular docking results: binding energies (MolDock score) and re‐rank scores for the interactions between ligands and mosquito odorant‐binding proteins.

Targets	Ligands	MolDock score (kcal/mol)	Re‐rank score (kcal/mol)	HBond
AaegOBP1	Carvacrol	−78.0	−67.1	−2.3
	Thymol	−78.7	−67.4	−2.9
	Cinnamaldehyde	−71.4	−62.0	0
	DEET	−85.8	−67.2	0

AgamOBP1	Carvacrol	−78.3	−68.5	−1.5
	Thymol	−76.8	−66.0	−2.7
	Cinnamaldehyde	−69.7	−61.3	−0.3
	DEET	−84.7	−74.7	0

AgamOBP5	Carvacrol	−80.6	−55.7	−4.5
	Thymol	−78.9	−45.1	−2.5
	Cinnamaldehyde	−70.2	−59.5	−0.5
	DEET	−81.0	−53.9	−1.8

AgamOR1	Carvacrol	−80.3	−48.5	−2.6
	Thymol	−77.52	−45.9	−2.2
	Cinnamaldehyde	−67.8	−58.3	−1.9
	DEET	−78.4	−59.2	0

AsteOBP1	Carvacrol	−74.1	−65.01	−2.2
	Thymol	−73.4	−64.2	−2.4
	Cinnamaldehyde	−68.0	−59.5	−0.9
	DEET	−84.4	−66.9	0

### 3.2. Sequential Docking

**FIGURE 11:**
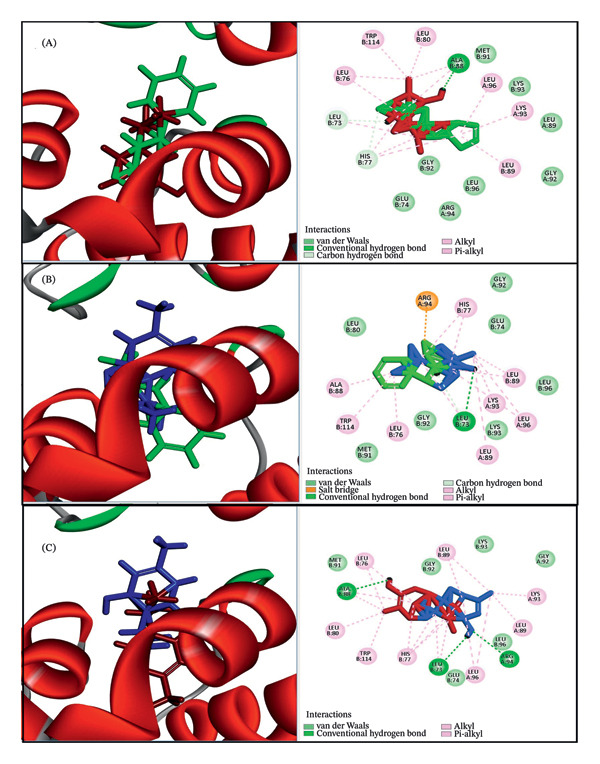
Sequential docking of ligand pairs in AaegOBP1, where the second ligand is docked onto the preformed ligand–protein complex: (A) cinnamaldehyde with carvacrol–OBP1 complex, (B) cinnamaldehyde with thymol–OBP1 complex, and (C) thymol with carvacrol–OBP1 complex. Cinnamaldehyde: green; carvacrol: red; thymol: blue.

**FIGURE 12:**
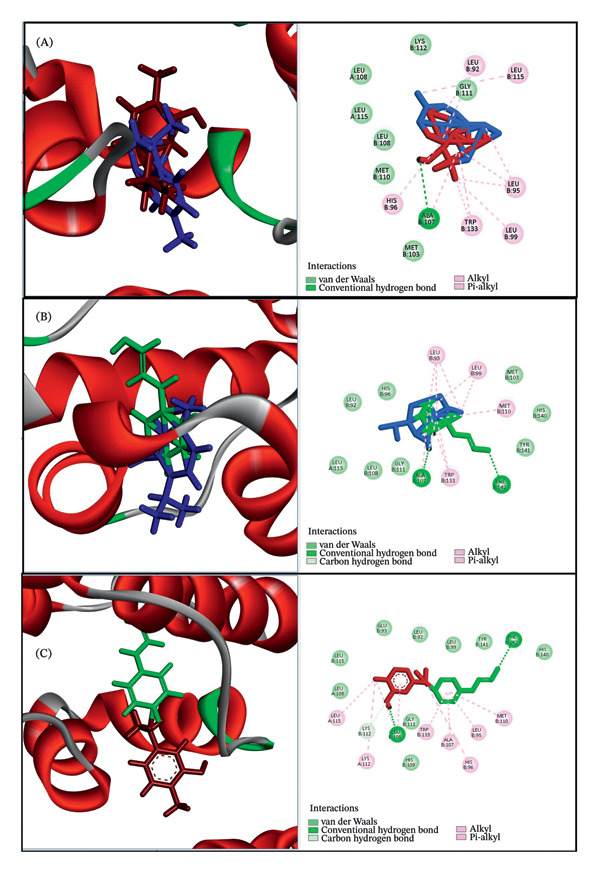
Sequential docking of ligand pairs in AsteOBP1, where the second ligand is docked onto the preformed ligand–protein complex: (A) thymol with carvacrol–OBP1 complex, (B) thymol with cinnamaldehyde–OBP1 complex, and (C) carvacrol with cinnamaldehyde–OBP1 complex. Cinnamaldehyde: green; carvacrol: red; thymol: blue.

Sequential docking analysis of both targets (AaegOBP1 and AsteOBP1) revealed that the simultaneous binding pattern of the compounds was strongly influenced by their structural similarity or dissimilarity. In cases where the ligands were chemically distinct, such as cinnamaldehyde in combination with thymol or carvacrol, simultaneous occupancy of adjacent but distinct regions of the binding cavity was possible without complete steric overlap or undesirable steric clashes. This type of complementary binding was accompanied by an expansion of the network of hydrophobic and polar interactions and could provide a suitable structural explanation for the hypothesized complementary or additive action. In contrast, compounds with high structural similarity, such as thymol and carvacrol, which are positional isomers with almost identical chemical skeletons, showed a partially overlapping binding pattern, indicating occupancy of a common site and the possibility of competition or additive effects. Overall, these results suggest that the complementary binding behavior of the two targets is more dependent on structural differences, complementary occupancy of subsites, and the increased total interactions within the binding cavity than on direct ligand overlap (Figures 11 and 12).

## 4. Discussion

This exploratory pilot study demonstrated that the thymol/carvacrol/cinnamaldehyde‐loaded nanogel provided prolonged mosquito repellency against both mosquito species. The nanogel achieved a mean protection time of 410 ± 45 min against *An. stephensi*, significantly outperforming DEET (40%), whereas it provided 220 ± 17 min of protection against *Ae. aegypti*, which was comparable to DEET (40%) [30]. These findings are consistent with previous reports on essential oil‐based nanoformulations, including *Mentha piperita* and *Eucalyptus globulus* nanoemulsions, which provided protection for 257–351 min against *An. stephensi* [31, 32]. Likewise, nanoemulsions prepared from *Cymbopogon nardus*, *Ficus glomerata*, and citronella oils have been reported to provide 168–234 min of protection against *Ae. aegypti* [33, 34].

The shorter protection time observed against *Ae. aegypti* compared with *An. stephensi* likely reflects species‐specific differences in susceptibility to repellents. *Ae. aegypti* possesses more efficient metabolic detoxification mechanisms, including cytochrome P450‐mediated oxidation, esterases, glutathione S‐transferases, and modified cuticular hydrocarbons that reduce compound penetration. In addition, differences in olfactory receptor sensitivity and behavioral responses may contribute to the greater tolerance of this species to botanical repellents [35, 36].

HPMC was selected as the gelling agent instead of conventional carboxymethyl cellulose (CMC) because of its superior aqueous solubility, thermal stability, and mechanical integrity, which contribute to improved formulation stability under diverse environmental conditions [37, 38]. Its adjustable viscosity allows precise rheological control and sustained release of volatile constituents, while its inverse thermoreversible behavior provides additional formulation flexibility [39, 40]. Furthermore, HPMC is biocompatible and nonirritating, and forms smooth, nongreasy gels that improve user acceptability while protecting volatile essential oils against oxidative degradation [41, 42]. Compared with CMC, HPMC also exhibits greater pH and thermal stability, superior film‐forming properties, and lower ionic character, thereby minimizing unfavorable interactions with encapsulated phytochemicals and supporting sustained release [43, 44].

The enhanced repellency observed for the nanogel may be attributed to the complementary biological activities of thymol, carvacrol, and cinnamaldehyde together with the sustained‐release characteristics of the HPMC nanogel matrix. Although synergistic interactions are plausible, they were not directly evaluated in the present study and therefore remain hypothetical. Thymol has been reported to achieve 99.5% landing inhibition in *Ae. aegypti* through behavioral repellency [45, 46]. In addition to its olfactory effects, thymol blocks GABA‐gated chloride channels, disrupts neural transmission, and induces larvicidal and adulticidal activity through hemocyte necrosis [47, 48]. Carvacrol, which is structurally related to thymol, interacts with OBPs involved in host detection, as demonstrated in *An. gambiae* [49]. Previous studies have also reported additive or potentially synergistic interactions between carvacrol and thymol together with low mammalian cytotoxicity [50, 51]. Cinnamaldehyde further complements these activities by modulating sensory and ionotropic receptors, including interactions with residues such as ASN218 in chemosensory proteins [52]. Moreover, inhibition of acetylcholinesterase and possible modulation of calcium channels may contribute to mosquito neuromuscular impairment [53, 54]. Incorporation of these phytochemicals into nanoformulations may enhance the persistence of their biological activity, as previously demonstrated for cinnamaldehyde‐based nanosystems that significantly prolonged in vivo repellency [55].

Encapsulation of the active phytochemicals within the HPMC nanogel is expected to improve skin adherence, increase residence time, and provide sustained release of volatile constituents. The non‐Newtonian rheological behavior described by the Carreau–Yasuda model enhances spreadability while maintaining prolonged release [56, 57]. Furthermore, the nanogel forms a surface reservoir that prolongs protection while minimizing dermal penetration and potentially reducing the risk of skin irritation compared with conventional DEET‐based formulations [58, 59].

*In silico* analyses supported these findings, showing that carvacrol and thymol bind strongly to mosquito olfactory receptors, with affinities comparable to or exceeding those of DEET, via hydrogen bonding and hydrophobic interactions. As shown in Table 2, the docking scores for carvacrol/thymol reached values as low as −78.0 and −78.7 kcal/mol, respectively, in the MolDock system. These high negative scores indicate a strong binding affinity, consistent with the favorable binding energy range (e.g., −6.4 to −5.4 kcal/mol) reported for related botanical compounds like α‐pinene and linalool by Okoli et al. using other molecular docking software [60]. Both studies identified conserved binding residues (Leu73, Leu76, Ala88, Met91, Lys93, Trp114), indicating a structurally conserved binding pocket representing a promising molecular target for plant‐based repellents.

Sequential docking of OBP1 from *Ae. aegypti* and modeled OBP1 from *An*. *stephensi* showed that in compounds with greater structural difference, binding of the second ligand to the preformed ligand–protein complex can occur without complete spatial overlap or adverse steric interference, providing a plausible structural explanation for the hypothesized complementary or additive action. In contrast, compounds with high structural similarity showed a more overlapping binding pattern in stepwise docking, suggesting the possibility of competitive or additive effects rather than true synergism.

These results highlight the potential of plant‐derived nanogels as effective, nontoxic alternatives to synthetic repellents, addressing increasing health and environmental concerns. Although further validation under field conditions is warranted, this work provides a strong foundation for developing next‐generation botanical mosquito repellents.

## 5. Limitations

This study has several limitations. As an exploratory pilot investigation, the biological evaluation was conducted using a single volunteer, and the reported triplicates represent technical rather than biological replicates. Consequently, biological replication was not achieved, interindividual variability could not be assessed, and the findings should not be generalized without caution. Furthermore, repellency was evaluated under controlled laboratory conditions against only two mosquito species. Therefore, future studies involving larger cohorts of volunteers, biological replication, and field evaluations are required to confirm the reproducibility and generalizability of the present findings.

## 6. Conclusion

This exploratory pilot study demonstrated that a multicomponent herbal nanogel containing thymol, carvacrol, and cinnamaldehyde provides prolonged mosquito repellency against both *Ae. aegypti* and *An. stephensi*. The nanogel significantly outperformed DEET (40%) against *An. stephensi* and showed comparable efficacy against *Ae. aegypti*. Molecular docking provided supportive mechanistic evidence but did not establish biological synergism. These preliminary findings support the potential of plant‐based nanogels as mosquito repellents and warrant validation through biological replication, larger cohorts of volunteers, and field studies.

## Author Contributions

Mohsen Safaei performed in silico studies. Alireza Sanei‐Dehkordi: bioassay execution. Navid Alinejad revised the manuscript. Mohsen Safaei and Mahsa Fallahi prepared nanogels. Mahmoud Osanloo: conceptualization, data analysis, and manuscript revision. All authors contributed to the drafting of the manuscript.

## Funding

This work was supported by Fasa University of Medical Sciences (Grant No. 403109).

## Disclosure

All authors approved the final version.

## Ethics Statement

This investigation evaluated a herbal nanogel against DEET, in accordance with WHO protocols. Per Iranian national guidelines, the study was exempt from classification as a clinical trial. After explaining the procedures in detail, the 22‐year‐old female volunteer provided written informed consent. Fasa University of Medical Sciences (IR.FUMS.REC.1404.020) granted ethical approval. Medical monitoring was provided throughout the study and 30 days post‐trial, with no adverse effects reported.

## Consent

The authors have nothing to report.

## Conflicts of Interest

The authors declare no conflicts of interest.

## Data Availability

All datasets supporting this study are included in the manuscript.
